# Detection of vector-borne pathogens in cats and their ectoparasites in southern Italy

**DOI:** 10.1186/s13071-016-1534-1

**Published:** 2016-05-10

**Authors:** Maria-Flaminia Persichetti, Laia Solano-Gallego, Lorena Serrano, Laura Altet, Stefano Reale, Marisa Masucci, Maria-Grazia Pennisi

**Affiliations:** Istituto Zooprofilattico Sperimentale della Sicilia Adelmo Mirri, Via G. Marinuzzi 3, 90129 Palermo, Italy; Departament de Medicina i Cirurgia Animal, Facultat de Veterinària. Universitat Autònoma de Barcelona, 08193 Bellaterra Barcelona, Spain; Vetgenomics, Edifici Eureka, PRUAB, 08193 Bellaterra Barcelona, Spain; Dipartimento di Scienze Veterinarie, Università di Messina, Polo Universitario Annunziata, 98168 Messina, Italy

**Keywords:** Vector-borne pathogens, Flea, Tick, Cat

## Abstract

**Background:**

Vector-borne pathogens are the subject of several investigations due to the zoonotic concern of some of them. However, limited data are available about the simultaneous presence of these pathogens in cats and their ectoparasites. The aim of the present study was to define the species of ectoparasites found on cats as well as to investigate vector-borne pathogens in cats and their ectoparasites in southern Italy.

**Methods:**

Blood from 42 cats and fleas or flea pools (*n* = 28) and ticks (*n* = 73) collected from them were investigated by quantitative PCR for the detection of vector-borne pathogens. Feline serum samples were tested by IFAT to detect IgG antibodies against *Leishmania infantum, Bartonella henselae*, *Rickettsia conorii, Rickettsia felis, Rickettsia typhi*, *Babesia microti*, *Ehrlichia canis* and *Anaplasma phagocytophilum* antigens.

**Results:**

Only one flea species (*Ctenocephalides felis*) and four tick species belonging to the genera *Rhipicephalus* and *Ixodes* were identified on cats from southern Italy. Molecular evidence of *Bartonella* spp., *Rickettsia* spp., hemoplasmas, *Babesia vogeli* and *L. infantum* was found in ectoparasites (fleas and/or ticks) while DNA from *Hepatozoon felis* and *Ehrlichia/Anaplasma* spp. was not detected. Likewise, DNAs from *Bartonella,* hemoplasma and *Leishmania* were the only pathogens amplified from feline blood samples. Cats had also antibodies against all the investigated pathogens with the exception of *Rickettsia typhi*. Agreement between serological and molecular results in individual cats and their ectoparasites was not found. The only exception was for *Bartonella* with a fair to moderate agreement between individual cats and their ectoparasites. *Bartonella clarridgeiae* was the species most frequently found in cats and their fleas followed by *B. henselae*.

**Conclusions:**

In conclusion, cats harboring ticks and fleas are frequently exposed to vector-borne pathogens. Furthermore, ticks and fleas harbored by cats frequently carry pathogens of zoonotic concern therefore appropriate feline ectoparasiticide preventative treatments should be used in cats.

## Background

Ticks, fleas and mosquitoes are globally distributed and their ability to transmit pathogens gives them important medical relevance. On the other hand, the growing success of pets in developed countries, especially the cat with its independent lifestyle, results in an increased risk for humans of contact with feline ectoparasites [1]. It is well known that the most frequent flea species found on cats is *Ctenocephalides felis* [2]. Conversely, limited information is available about the species of ticks which infest cats and vector-borne pathogens (VBPs) harbored by them [2–10]. In addition, the comparison of vector-borne pathogens from cats and from their ectoparasites (fleas and ticks) has not been fully explored [11].

The aims of this investigation which was carried out in two regions (Calabria and Sicily) of southern Italy were: (i) to evaluate the flea and tick species collected from outdoor domestic cats and determine if they harbor VBPs; (ii) to evaluate exposure of outdoor cats to VBPs by means of antibody and molecular testing; and (iii) to compare the VBPs DNA from feline blood and from the ectoparasites (fleas and ticks) collected from them.

## Methods

The present study integrates data already published on 132 ticks collected from a large number of cats (*n* = 308) in Southern Italy and the pathogens that they harbor [4]. We included in this study a total of 42 cats from province of Reggio Calabria (*n* = 27) and from Messina city and Lipari Island in Sicily region (*n* = 15) enrolled between March 2012 and January 2013. These cats were selected based on the following criteria: the presence of at least one ectoparasite (tick or flea) on physical examination, residual ethylenediaminetetraacetic acid (EDTA) blood and serum samples available, signed owner informed consent and outdoor life style. Both sick (22/42 = 52.4 %) and apparently healthy cats (20/42 = 47.6 %) based on clinical history and physical examination were enrolled. Date of sampling, gender, age, breed, lifestyle, vaccination status, ongoing therapy, reason for consultation, physical examination, the number of collected ectoparasites, feeding status of collected ticks as well as antiparasitic treatments of cats were recorded.

Each cat was carefully combed for at least five minutes throughout the whole body surface and inspected for the presence of fleas or/and ticks. All ectoparasites detected were removed by a veterinarian and stored in alcohol 70 % as a preventative measure. Feline blood residual samples were employed in the present study. Therefore, ethical committee approval was not needed. Informed consent was obtained from all owners and from the legal representative of animal welfare groups in charge of the management of stray cats.

Serum from all cats was tested for the detection of immunoglobulin G (IgG) antibodies against *Bartonella henselae*, *Rickettsia conorii*, *Rickettsia felis*, *Rickettsia typhi, Ehrlichia canis*, *Babesia microti* and *Anaplasma phagocytophilum* antigens by the immunofluorescence antibody test (IFAT) using commercial kits (Fuller Laboratories Fullerton, California, USA). The manufacturer’s protocol was followed for all serological tests using a cut-off dilution of 1:64 for *B. henselae*, *R. conorii, R. felis, R. typhi* and *B. microti*; and 1:50 for *E. canis* and *A. phagocytophilum*. The presence of *L. infantum* IgG antibodies was investigated using *L. infantum* (strain MHOM/IT/80/IPT1) antigen slides manufactured by the *National reference centre for Leishmaniosis, (*C.Re.Na.L, Palermo, Italy) and fluoresceinated rabbit anti-cat IgG (Anti-IgG-FITC, SIGMA) diluted in PBS 1:200 [12]. The cut-off value was established at 1:80 for *L. infantum* [12].

Morphometric identification of fleas and ticks was made through a stereomicroscope before DNA extraction for polymerase chain reaction (PCR) assays [13, 14]. Afterwards, fleas from each cat were extracted and processed and only for cats carrying more than one single flea, pools were done. Specifically, a number spanning from two to five fleas collected from each cat was pooled for molecular investigations. Conversely, ticks were in any case extracted and processed individually.

DNA extraction from 300 μl of blood was performed using High Pure PCR Template preparation kit (Roche, Mannheim, Germany). DNA was eluted in 100 μL of elution buffer and stored at -20 °C until used. DNA extraction from individual ticks, fleas and flea pools was carried out using High Pure PCR Template preparation kit (Roche, Mannheim, Germany) according to the manufacturer’s tissue protocol with some modifications. Briefly, all ectoparasites were washed twice in sterile PBS solution for 5 min shaking it slowly, then overnight at 4 °C. Each flea was manually cut by a sterile lancet in four pieces and then suspended in 200 μl of Tissue Lysis Buffer of the same kit. DNA was eluted in 50 μl of elution buffer and stored at -20 °C for later analysis.

Real-time PCR technology was applied as described elsewhere [4], to identify specific DNA target for *Ehrlichia/Anaplasma* spp., piroplasmids (*Babesia* spp. and *Theileria* spp.), *Hepatozoon felis*, hemotropic *Mycoplasma* spp., *Rickettsia* spp., *Bartonella* spp. and *L. infantum* from ticks and feline blood samples while only the last four pathogens were investigated on fleas due to economical restrictions. All positive PCR results for each ectoparasite or cat were sequenced according to the Big-Dye Terminator Cycle Sequencing Ready reaction Kit (AB, Life Technologies) using the same primers. Sequences obtained were compared with GenBank database (www.ncbi.nlm.nih.gov/BLAST). All positive PCR results for hemoplasmas or *L. infantum* were not sequenced. Instead, species-specific real time PCRs were performed as described by Martinez et al. [15] to discriminate among feline hemoplasmas species (*Mycoplasma haemofelis* (*Mhf*), ‘*Candidatus* Mycoplasma haemominutum’ (*C*Mhm) and ‘*Candidatus* Mycoplasma turicencis’ (*C*Mt)) as well as for *L. infantum* real time PCR [16].

For each pathogen investigated, Kappa agreement test (GraphPad InStat) was used to establish agreement between serological and molecular results in cats, between molecular results in cats, ticks or fleas and between serological results in cats and molecular results in ticks or fleas. The Kappa values were evaluated as follows: no agreement (*k* < 0), slight agreement (0 < *k* < 0.2), fair agreement (0.2 < *k* < 0.4), moderate agreement (0.41 < *k* < 0.6), substantial agreement (0.61 < *k* < 0.8) and almost perfect agreement (*k* > 0.81).

## Results

### Clinical data and antibody detection in cats

Age of cats ranged from six months to ten years with a median of 1.5 years. Twenty-three cats were females and 19 were males. Only six were not neutered. Thirty-nine were mixed breed domestic short hair cats and six mixed breed domestic long hair cats. Information on ectoparasiticide treatment was available for 40 cats and most of them (35/40 = 87.5 %) were never treated with ectoparasiticide. One cat was only treated during the summer season but the other four cats were monthly treated. Ticks were also detected on these five treated cats.

Thirty-nine of the 42 cats examined (92.9 %) were antibody positive to at least one investigated antigen. Antibodies were detected against all the VBPs studied except for *R. typhi* antigens (Table 1).

**Table 1:** Serological results of investigated pathogens in 42 cats infested by ectoparasites

Antigen	Number of seropositive cats (%)
*Bartonella henselae*	23 (54.8)
*Rickettsia conorii*	23 (54.8)
*Anaplasma phagocytophilum*	14 (33.3)
*Babesia microti*	10 (23.8)
*Ehrlichia canis*	6 (14.3)
*Leishmania infantum*	1 (2.4)
*Rickettsia felis*	1 (2.4)
*Rickettsia typhi*	0 (0)

### Detection and morphological identification of ticks and fleas

Sixty-ffwas infested by both ticks and fleasive fleas were collected from 28 out of the 42 cats and all were identified as *C. felis*. Seventy-three ticks were also removed from 15 cats and only one cat (from Calabria) was infested by both ticks and fleas. Ticks belonged to the genera  *Rhipicephalus* (*n* = 42) and *Ixodes* (*n* = 31) and 25 specimens were engorged. In detail, *25 Rhipicephalus sanguineus* (3 engorged), 17 *Rhipicephalus pusillus,* 19 *Ixodes ventalloi* (16 engorged), ten *Ixodes ricinus* (four engorged), two engorged *Ixodes * spp. were identified. The number of ectoparasites collected from individual cats ranged from one to five fleas with a median value of two fleas and one to 21 ticks with a median value of four ticks. Fleas were mostly (27/28 = 96 %) collected from cats in Calabria Province with the exception of one male flea that was removed from a cat living in Messina city. In contrast, all ticks were found on cats from Lipari island (Messina province) (14/15 = 93 %) with the exception of one tick *(Ixodes ricinus* engorged female) that was removed from a cat living in Calabria Province (1/15 = 7 %).

### Molecular results on ticks, fleas and feline blood samples

Almost all fleas (96.4 %), 19.2 % of ticks and 42.8 % of cats were found PCR-positive to at least one investigated pathogen. PCR results from ticks, fleas and cats are summarized in Table 2.

**Table 2:** Results of pathogens investigated by PCR in ticks, fleas and cats

Pathogens	Number of positive ticks, fleas or cats/total number of ticks, fleas or cats (%)		
	Ticks	Fleas^a^	Feline blood
*Bartonella* spp.	2/73 (2.7)	20/28 (71.4)	16/42 (38.1)
Hemoplasmas	0 (0)	0 (0)	11/42 (26.2)
*Rickettsia* spp.	5/73 (6.8)	23/28 (82.1)	0 (0)
*Ehrlichia* spp./*Anaplasma* spp.	0 (0)	NE	0 (0)
Piroplasmid	1/73 (1.4)	NE	0 (0)
*Hepatozoon felis*	0 (0)	NE	0 (0)
*Leishmania infantum*	8/73 (10.9)	2/28 (7.1)	3/42 (7.1)
Total (%)^b^	14/73 (19.2)	27/28 (96.4)	18/42 (42.8)

*NE:* not evaluated

^a^10 single fleas and 18 flea pools (range 2–5 fleas/pool); ^b^Total number of specimens positive at least to one pathogen. Co-infections were counted only once

*Bartonella clarridgeia*e was confirmed by sequencing in seven cats (16.6 %), in 16 pools of fleas (57.1 %) and in two ticks (1.5 %). *Bartonella henselae* was confirmed by sequencing in nine cats (21.4 %) and in only four pools of fleas (14.3 %). DNA sequences were 99–100 % identical to both *Bartonella* species available in GenBank (*B. clarridgeiae* (GenBank ID: FN645454.1) and *B. henselae* (GenBank ID: KF466255.1). Similarly, *L. infantum* DNA was amplified from three cats, two fleas or pools and eight ticks.

Interestingly, DNA of hemoplasmas was not amplified from any of the ectoparasites but 11 feline blood samples were positive. Briefly, seven *Mhf*, seven *C*Mhm and four *C*Mt DNAs were detected in cats. *Babesia vogeli* was only amplified from one tick with a 100 % identity of GenBank sequences (GenBank ID: JX871885.1).

*Rickettsia monacensis* and *R. helvetica* were detected in five ticks and DNA sequences were 98–100 % identical to GenBank sequences (*R. monacensis* (GenBank ID: KF016136.1) and *R. helvetica* (GenBank ID: JQ796866.1). *Rickettsia felis* was found in 23 flea pools or single fleas with an identity of 100 % of GenBank sequences (GenBank ID: KF245441.1).

Six out of 25 engorged ticks were PCR positive to at least one investigated pathogen but they never harbored the same microorganisms of the host cat. Individual results of serology and PCR from cats and their ectoparasites are listed in Tables 3 and 4. In feline blood, the most frequent co-infection was among different hemoplasma species (*n* = 6). Moreover, co-infection was found between *Bartonella* spp. and hemoplasmas (*n* = 3). *Leishmania infantum* DNA was also amplified in one cat positive to both *B. henselae* and *B. clarridgeiae*.

**Table 3:** Distribution of serological and PCR results in cats and in their ticks

Number of cats	Pathogens exposure confirmed by antibody detection in cats	Pathogens identified by real-time PCRs	
		Cat	Ticks
1	*R. conorii, B. microti*	None	None
2	*R. conorii, A. phagocytophilum*	None	None
1	*B. henselae, R. conorii, E. canis, A. phagocytophilum*	*L. infantum*	None
1	*B. henselae, R. conorii, A. phagocytophilum*	*B. henselae*, *C*Mhm	None
1	*B. henselae, B. microti*	*B. henselae*	None
1	*R. conorii, A. phagocytophilum*	*Mhf*	None
1	*R. conorii, B. microti*	None	*L. infantum*
1	*B. henselae, B. microti*	*B. henselae*	*L. infantum*
1	*B. henselae, R. conorii, B. microti*	*C*Mhm, *C*Mt	*B. clarridgeiae*
1^b^	Negative	*Mhf, C*Mhm	*R. monacensis*
1	*A. phagocytophilum*	*B. clarridgeiae*	*R. monacensis,* *L. infantum*
1	*B. microti*	None	*B. vogeli* *L. infantum*
1	*B. henselae, R. conorii, A. phagocytophilum*	*B. henselae, Mhf, C*Mhm	*B. clarridgeiae,* *L. infantum*
1^a^	*R. conorii, R. felis, E. canis, B. microti*	None	*R. helvetica*, *R. monacensis*, *L. infantum*

Abbreviations: *Mhf Mycoplasma haemofelis*, *CMhm Candidatus* Mycoplasma haemominutum, *CMt Candidatus* Mycoplasma turicensis

^a^Cat infested by 21 ticks; ^b^ Cat infested by both ticks and fleas

**Table 4:** Distribution of serological and PCR results in cats and in their fleas

Number of cats	Pathogens exposure confirmed by antibody detection in cats	Pathogens identified by real-time PCRs	
		Cats	Fleas
1	*B. henselae*	None	None
1	*B. henselae*	None	*B. henselae*
1	Negative	*Mhf, B. clarridgeiae*	*B. clarridgeiae*
2	*B. henselae*	*B. henselae*	*B. clarridgeiae*
1	Negative	None	*R. felis*
1^a^	Negative	*Mhf, C*Mhm	*R. felis*
1	*R. conorii*	None	*R. felis*
1	*B. henselae*	*C*Mhm, *C*Mt	*R. felis*
1	*B. henselae, R. conorii*	*C*Mhm, *C*Mt	*R. felis*
1	*R. conorii,* *A. phagocytophilum*	None	*R. felis*
1	*B. henselae, R. conorii*	*B. henselae,* *L. infantum*	*B. henselae, R. felis*
1	*B. henselae*	*B. clarridgeiae*	*B. henselae, R. felis*
1	*B. henselae,* *A. phagocytophilum*	*B. henselae*	*B. henselae, R. felis*
2	*R. conorii*	None	*B. clarridgeiae,* *R. felis*
1	*A. phagocytophilum*	None	*B. clarridgeiae,* *R. felis*
1	*B. henselae, R. conorii, E. canis, B. microti,* *L. infantum*	None	*B. clarridgeiae,* *R. felis*
1	*B. henselae, B. microti*	*Mhf, C*Mhm, *C*Mt	*B. clarridgeiae,* *R. felis*
1	*R. conorii*	*Mhf*	*B. clarridgeiae,* *R. felis*
1	*B. henselae, R. conorii*	*B. henselae*	*B. clarridgeiae,* *R. felis*
1	*B. henselae, E. canis*	*B. clarridgeiae*	*B. clarridgeiae,* *R. felis*
1	*B. henselae, R. conorii, E. canis, A. phagocytophilum*	*B. clarridgeiae*	*B. clarridgeiae,* *R. felis*
1	*B. henselae, B. microti*	*B. clarridgeiae*	*B. clarridgeiae,* *R. felis*
1	*B. henselae*	*Mhf*	*B. clarridgeiae,* *R. felis*
1	*A. phagocytophilum*	*B. clarridgeiae,* *L. infantum*	*B. clarridgeiae,* *R. felis*
1	*R. conorii,* *A. phagocytophilum*	None	*R. felis, L. Infantum*
1	*B. henselae, R. conorii, E. canis, A. phagocytophilum*	None	*B. clarridgeiae,* *R. felis, L. Infantum*

Abbreviations: *Mhf Mycoplasma hemofelis*, *CMhm Candidatus* Mycoplasma haemominutum, *CMt Candidatus* Mycoplasma turicensis

^a^Cat infested by both ticks and fleas

Molecular investigations detected DNA of different pathogens in single ticks. Briefly*, L. infantum* DNA was amplified in three ticks already positive to *B. clarridgeiae*, *R. monacensis* and *B. vogeli* and another tick was found positive to both *R. monacensis* and *R. helvetica.*

In fleas, the most frequent co-infection was between *R. felis* and *B. clarridgeiae* that was found in pools but also in one single flea. Only four fleas, collected from a PCR negative cat, were positive at the same time to three pathogens (*R. felis*, *B. clarridgeiae* and *L. infantum*)*.*

The agreement between serological *Bartonella* results in cats and PCR data was moderate with ticks (*k* = 0.461; accuracy 0.79), followed by a fair agreement with feline blood (*k* = 0.395; accuracy 0.69) and with fleas (*k* = 0.292; accuracy 0.68). A fair or slight agreement was respectively observed between *B. henselae* PCR results in cats and in fleas (*k* = 0.340; accuracy 0.82) as well as for *B. clarridgeiae* (*k* = 0.208; accuracy 0.57). Agreement between serological and molecular results for other pathogens in individual cats and their ectoparasites was slight or not found.

## Discussion

In this study, we confirmed *C. felis* as the unique flea species found in cats from Southern Italy mainly in Calabria Province. In contrast, species of *Rhipicephalus* and *Ixodes* were found as the only tick species collected from cats mainly living in Lipari Island (Sicily) [4]. Ecological factors, season of sampling, climatic variations may be responsible for these findings. Moreover, outdoor cats from Lipari Island are free roaming in a wild habitat and therefore they are in close contact with wild rabbits and birds and their ectoparasites.

Almost all fleas (96.4 %), 19.2 % of ticks and 42.8 % of cats were found PCR positive to at least one investigated pathogen. The most common pathogens identified by molecular techniques were of zoonotic concern and include *Rickettsia*, *Bartonella* and *L. infantum*, however with different distribution and rate of infection in cats and their ectoparasites. For instance, *Rickettsia* spp. DNA was exclusively amplified from the ectoparasites (*R. helvetica* and *R. monacensis* from ticks and *R. felis* from fleas) supporting a possible role for cofeeding transmission in the maintenance of these pathogens within the vector population as already demonstrated for *R. conorii israelensis* in *R. sanguineus* ticks [17] and for *R. felis* in fleas (*C. felis* and *Xenopsilla cheopis*) [18]. These rickettsial species can cause febrile illness among other clinical manifestations in humans as well established for *R. felis* [19] and also described for *R. helvetica* and *R. monacensis* [20]*.* Therefore, it is important to highlight that cats will be carriers of ectoparasites and associated *Rickettsia* species to humans suggesting a zoonotic potential but they do not appear to be reservoirs of these infections. Conversely, *Bartonella* DNA (*B. clarridgeiae* and *B. henselae*) was the most frequent pathogen found in cats suggesting an important zoonotic risk to humans [21] as carriers of ectoparasites and apparent reservoirs for both infections [22]. Moreover, hemoplasma (*Mhf,* CMt*, C*Mhm) DNA was detected only in cat blood confirming the potential limited role of vectors in their transmission [23, 24] despite DNA of some hemotropic mycoplasmas can be found in ectoparasites collected from cats [11, 25, 26].

It is noteworthy that *L. infantum* DNA was found in 7–10 % of ectoparasite or cat blood samples and it was the most common parasite found in ticks. Interestingly, this is the first *bona fide* report of *L. infantum* DNA from cat fleas. DNA from *Bartonella henselae, B. clarridgeiae, R. monacensis, R. helvetica, R. felis, M. haemofelis, Ca.* M. haemominutum*, Ca.* M. turicensis, *B. vogeli* and *L. infantum* was amplified in feline blood and/or in ectoparasites. Vector-borne pathogens found in this study partly confirm previous data reported in Italy from cats or their ectoparasites [8–10, 27, 28]. However, we obtained data at the same time from cats and the ticks and fleas they harbor.

Detection of antibodies against most of the investigated VBPs and/or of circulating pathogen DNA showed that cats harboring ticks or fleas are frequently exposed to VBPs in the geographic area under study. In fact as much as half of cats had antibodies against *R. conorii* and *B. henselae* and the percentage of detectable antibodies against *A. phagocytophilum, B. microti* and *E. canis* was not negligible confirming data previously reported in Italy [10] and throughout Europe [6, 29–31]. Interestingly, in the present study, a very low *R. felis* antibody rate was observed (2.4 %) as well as no detection of *R. felis* DNA in any feline blood samples as reported in other studies [26, 32, 33]. Our findings are in disagreement with other data that reported higher *R. felis* antibody rates (16.3 %) in Spain [33] as well as *R. felis* DNA detection (28 %) in feline blood from Bangladesh cats [34]. In the present study, high *R. conorii* antibody rates were found in the absence of rickettsiemia as previously reported in other studies in cats [29]. Similar findings are also observed in dogs with high *R. conorii* antibody rates and low rickettsiemia in endemic areas [35]. However, the present findings are in disagreement with a study performed in northeastern Spain where *Rickettsia* DNA similar to *R. conorii* or *R. massiliae* was found in 10 % of cats [36]. Obviously, we cannot exclude that infections caused by other *Rickettsia* species of the spotted fever group circulating in southern Italy such as *R. massiliae* among others, contributed to this high antibody prevalence [36, 37]. It is well known that cross-reactions are common among *Rickettsia* species and this is a limitation of antibody prevalence studies [38]. Sicily and Calabria are Italian regions with a high incidence of human rickettsial diseases [39] and it is of peculiar interest to clarify the role of cats in their eco-epidemiology. In contrast with Spanish data [40], no serological and molecular traces of *R. typhi* were observed in both cats and ectoparasites.

Agreement between the majority of pathogens based on molecular or antibody detection among cats and in their ectoparasites was not found. The only exception was for fleas which were DNA positive to *Bartonella* (four for *B. henselae* and five to *B. clarridgeiae*). These fleas were collected from cats which were antibody and/or PCR positive to the same pathogen. Lappin et al. reported that almost all cats (94.7 %) infected by *B. clarridgeiae* carried *B. clarridgeiae* infected fleas and they suggested that *C. felis* may be a vector for this pathogen [11]. Conversely, in Taiwan, researchers found a high prevalence of fleas PCR positive for *B. clarridgeiae* and a very low prevalence of this bacterium in cats. They raised the hypothesis that *B. clarridgeiae* is more adapted to the flea than to the vertebrate host [5]. Other studies found a higher prevalence of *Bartonella* and hemoplasma DNA in fleas than in cat blood, but no data were given about positivity of fleas and their corresponding cat host [31, 41]. In this study, *B. clarridgeiae* was the microorganism most frequently found simultaneously in fleas and their feline host.

To the best of our knowledge, no studies compared the molecular detection of pathogens in ticks removed from cats and their host. Host molecular negativity for pathogens found in ectoparasites may depend on the vector competence. For tick-borne pathogens, the transmission occurs at different times after the beginning of the blood meal depending upon specific life cycle characteristics [24]. Moreover, the bacteremia of some VBPs is transient, lasting a few hours, as reported for *Rickettsia* spp. and *E. canis* in dogs. This makes it difficult to detect the pathogens in the blood stream [24]. The low level of circulating pathogens requires highly sensitive molecular tools.

## Conclusions

In conclusion, cats harboring ticks and fleas are frequently exposed to many VBPs. However, the simultaneous detection of VBPs in the hosts and their ectoparasites is uncommon with the exception of *Bartonella. Bartonella clarridgeiae* followed by *B. henselae* were the species most frequently found at the same time in fleas and the cat host.

Ticks and fleas harbored by cats frequently carry pathogens of zoonotic concern. As a preventative measure, the appropriate use of ectoparasiticide treatments is strongly recommended for use in cats.

## Abbreviations

°C: Celsius degree
Bp: base pairs
DNA: deoxyribonucleic acid
EDTA: ethylenediamine tetraacetic acid
IFAT: immunofluorescence antibody test
IgG: Immunoglobulins G
*k*: k agreement
min: minutes
*n*: number
OR: Odds Ratio
PBS: Phosphate buffered saline
PCR: Polymerase Chain Reaction
VBPs: Vector-Borne Pathogens
μL: microliters
